# Transcatheter embolization for hemoptysis associated with anomalous systemic artery in a patient with scimitar syndrome

**DOI:** 10.1186/s40064-015-1219-9

**Published:** 2015-08-14

**Authors:** Hideaki Yamakawa, Kanichiro Shimizu, Kenkichi Michimoto, Yoshihiko Kameoka, Ryeonshi Kang, Jun Yoshida, Masami Yamada, Masahiro Yoshida, Takeo Ishikawa, Masamichi Takagi, Kazuyoshi Kuwano

**Affiliations:** Division of Respiratory Medicine, Department of Internal Medicine, Kashiwa Hospital, Jikei University School of Medicine, 163-1 Kashiwashita, Kashiwa, Chiba, 277-8567 Japan; Department of Radiology, Kashiwa Hospital, Jikei University School of Medicine, Chiba, Japan; Division of Cardiology, Department of Internal Medicine, Kashiwa Hospital, Jikei University School of Medicine, Chiba, Japan; Division of Respiratory Medicine, Department of Internal Medicine, Jikei University School of Medicine, Tokyo, Japan

**Keywords:** Anomalous systemic arterial supply to the basal lung, Transcatheter embolization, Scimitar syndrome, Hemoptysis

## Abstract

**Background:**

Scimitar syndrome can present with a wide clinical spectrum of symptoms either early in the neonatal period or later in life.

**Case description:**

We report a case of a 62-year-old woman with anomalous systemic arterial supply to the basal lung with scimitar syndrome presenting as recurrent hemoptysis. Bronchoscopy revealed normal major bronchial branches without bronchial atresia, indicating that sequestration of the lung was not confirmed. The anomalous drainage of the scimitar vein was to the inferior vena cava, and an anomalous artery from the aorta supplied the right basal lung. There were no findings of pulmonary hypertension and arteriovenous malformation such as an anomalous artery to the scimitar vein. The distal portions of anomalous arteries were embolized using gelatin sponge particles and the proximal portion was embolized using fibered detachable coils. Although a small pulmonary infarction was observed as a complication, the patie nt has not experienced any subsequence recurrence of the hemoptysis during a follow-up period of 6 months.

**Discussion and evaluation:**

Deformities of the blood vessels and the lungs are frequently complex in scimitar syndrome. Although patients treated with surgical repair of this disorder may be at higher risk than those treated less invasively, we believe that transcatheter embolization was a useful strategy for the treatment of the anomalous systemic arterial supply to the basal lung, particularly in this patient with scimitar syndrome.

**Conclusion:**

Hemoptysis in a patient with scimitar syndrome associated with anomalous systemic arterial supply to the basal lung was successfully treated with transcatheter arterial embolization. However, it might be better to avoid the use of gelatin sponge particles in patients with a similar anomaly without pulmonary artery distribution because of the possibility of causing severe pulmonary infarction.

## Background

Scimitar syndrome, one of the large congenital pulmonary venolobar syndromes, is defined as a hypogenetic lung associated with partial anomalous pulmonary venous return to the inferior vena cava (IVC). Scimitar syndrome is further associated with hypoplasia of the right lung, congenital heart malformation, and anomalous systemic arterial supply to the lung. The presence of scimitar vein obstruction, the degree of the arterial supply to the lung, and the presence of bronchial abnormalities are responsible for retained bronchial secretions, lobar infections, and hemoptysis (Vida et al. 2009). We present a case of a patient with scimitar syndrome associated with anomalous systemic arterial supply to the right basal lung. The patient’s complaint of progressive and recurrent hemoptysis was successfully treated with transcatheter arterial embolization (TAE) of the anomalous systemic artery without surgical intervention. TAE of the systemic arterial supply may be a safe and feasible procedure to resolve hemoptysis in such cases.

## Case description

**Fig. 1:**
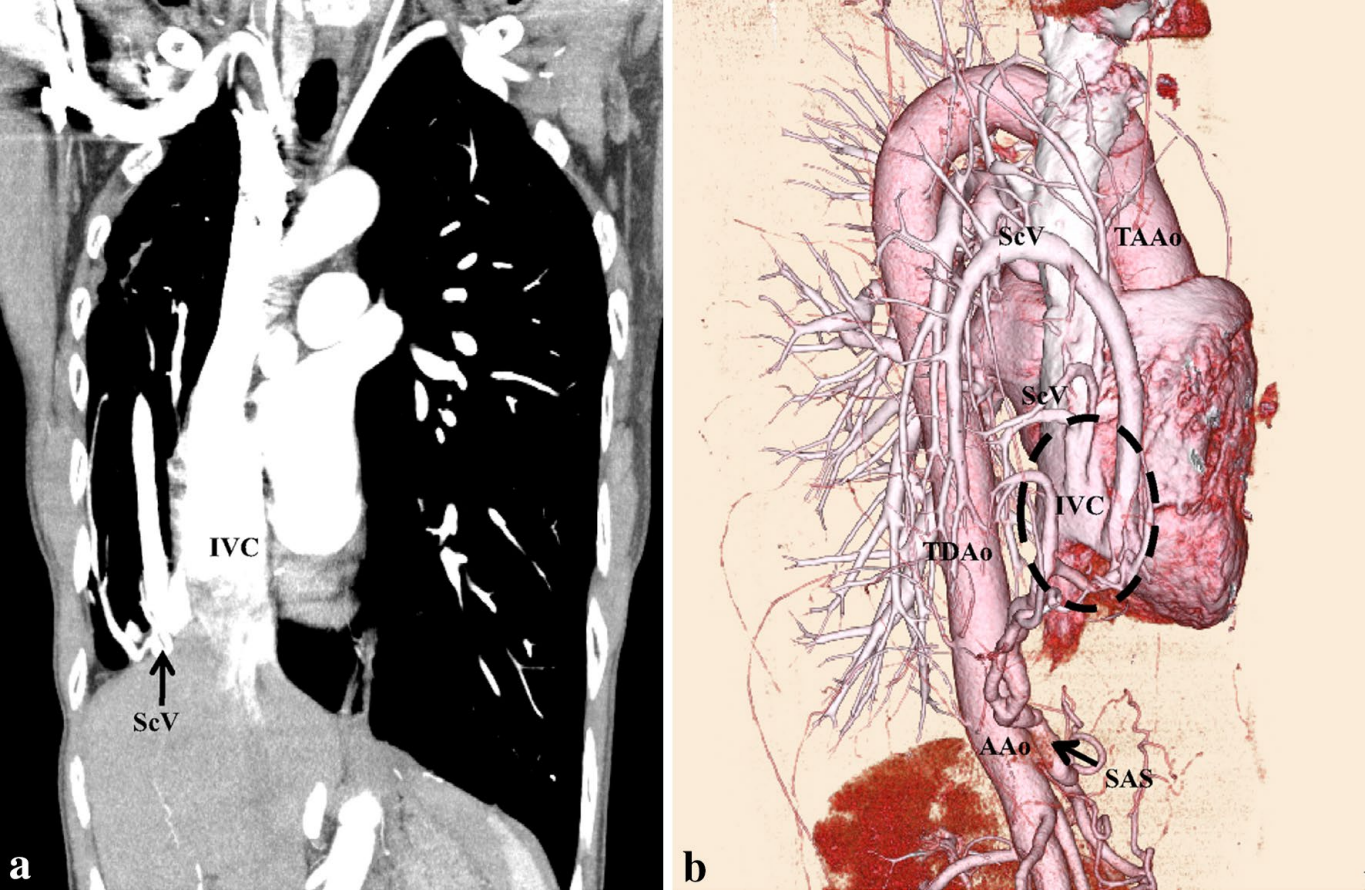
Coronal reconstruction of contrast-enhanced computed tomography showed scimitar vein drainage of the inferior vena cava (**a**). Three-dimensional volume-rendered computed tomography (*right anterior oblique view*) showed drainage of the inferior vena cava via several meandering scimitar veins (*dashed-line circle*) (**b**). *ScV* scimitar vein, *IVC* inferior vena cava, *TAAo* thoracic ascending aorta, *TDAo* thoracic descending aorta, *AAo* abdominal aorta, *SAS* systemic arterial supply

**Fig. 2:**
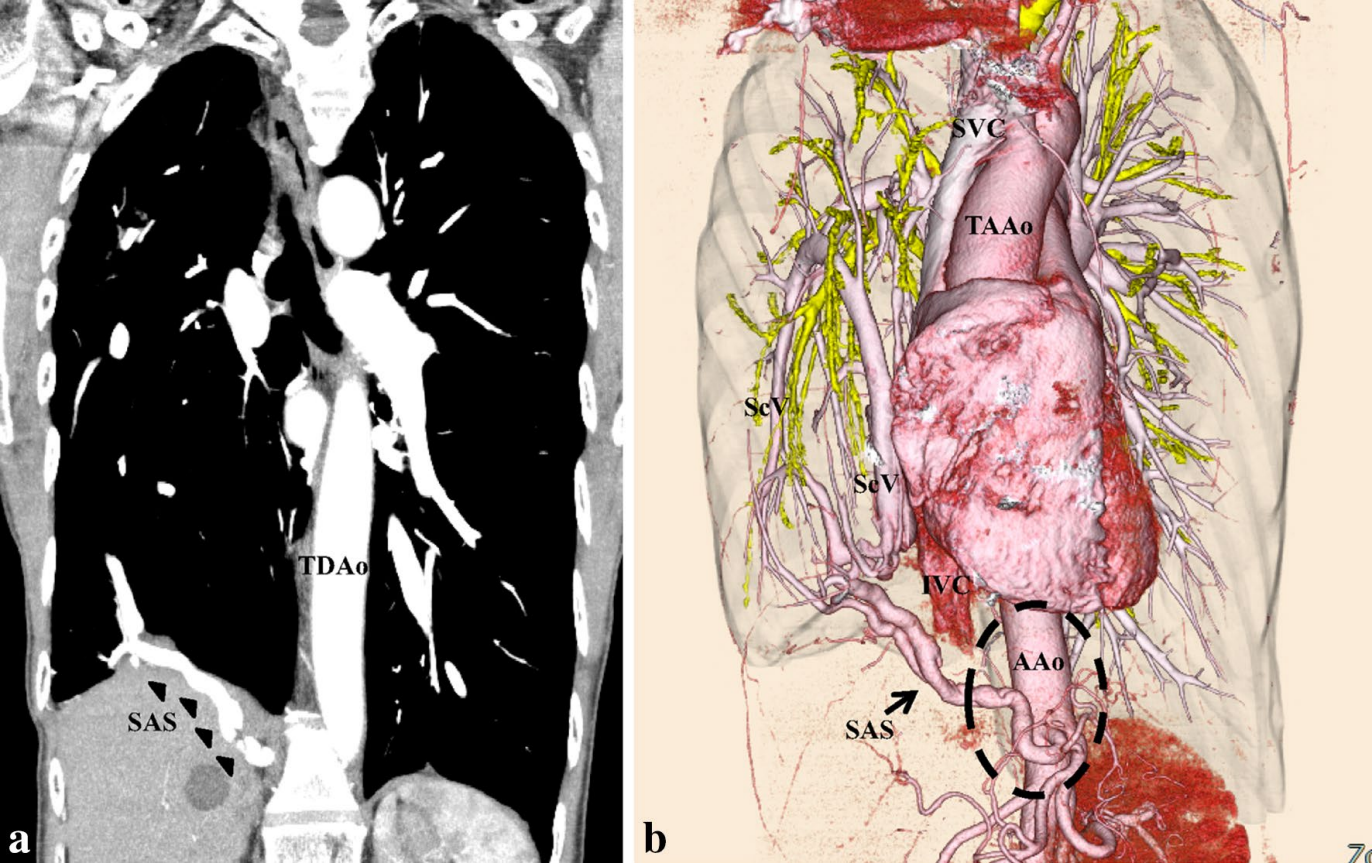
Coronal reconstruction of contrast-enhanced computed tomography showed the systemic arterial supply (*arrow*) to the right basal lung from the abdominal aorta (**a**). Three-dimensional volume-rendered computed tomography showed the meandering systemic arterial supply from the abdominal aorta (*dashed-line circle*) (**b**). *SAS* systemic arterial supply, *TDAo* thoracic descending aorta, *SVC* superior vena cava, *TAAo* thoracic ascending aorta, *ScV* scimitar vein, *IVC* inferior vena cava, *AAo* abdominal aorta

A 62-year-old woman with a 2-year history of slowly progressive, recurrent hemoptysis was referred to our hospital. On physical examination, she was afebrile and without dyspnea or hypoxemia. Chest radiography demonstrated a slightly opaque right hemithorax with nonvisualization of the heart, which was shifted to the same side, and a scimitar vein. Transthoracic echocardiography showed a mildly enlarged right atrium and right ventricle. Other findings, such as atrial and/or ventricular septal defect, were not confirmed. Color Doppler study showed the anomalous pulmonary vein draining into the IVC. Three-dimensional volume-rendered computed tomography (3DCT-VR) and multiplanar reformation with contrast material revealed three main findings as combined congenital anomalies of the lung and vascular system. First, the right lung was hypoplastic compared with the left lung, and bronchial deformation with dextroposition of the heart was present. Bronchoscopy revealed that the right main bronchus was obviously short, and the major bronchial branches were normal without bronchial atresia, indicating that sequestration of the lung was not confirmed. Second, the anomalous drainage of the right pulmonary veins (scimitar vein) was to the suprahepatic portion of the IVC (Fig. 1a, b). Third, an anomalous thick and tortuous artery, which originated from the aorta near the right inferior phrenic artery, supplied the right basal lung (Fig. 2a, b). The patient was diagnosed as having scimitar syndrome associated with anomalous systemic arterial supply to the right basal lung. Hemodynamic parameters obtained by right heart catheterization including a mean pulmonary capillary wedge pressure of 14 mmHg, pulmonary artery pressure of 30/12 (19) mmHg, and cardiac index of 3.73 L/min/m^2^, indicating no pulmonary hypertension. Her pulmonary-to-systemic blood flow ratio (Qp/Qs) was 1.88. An oxygen saturation step-up from the infrahepatic IVC to the right atrium was revealed (73.5 and 81.1 %, respectively). TAE of the anomalous systemic arterial supply was then planned.

**Fig. 3:**
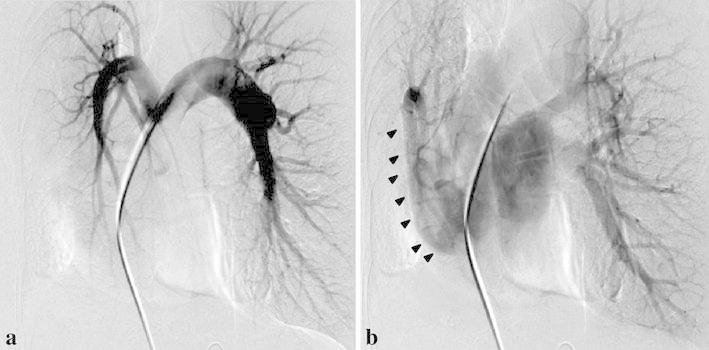
Pulmonary arteriography showed no contrast enhancement of the pulmonary artery in the right inferior lobe, revealing pulmonary artery hypoplasia (**a**). Contrast was returned to the right pulmonary vein (including the scimitar vein) (*arrowheads*) via the normal lung parenchyma (**b**)

**Fig. 4:**
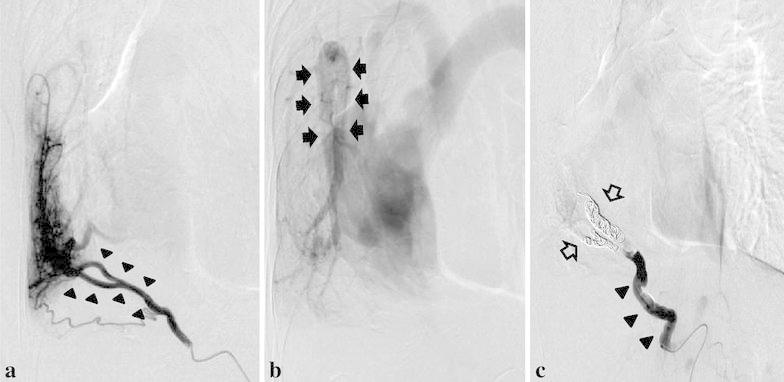
Selective angiography of the anomalous artery. The anomalous systemic artery supply (*arrowheads*) to the lung from the abdominal aorta supplied blood to a localized region of the right lower lobe (**a**). In a delayed phase, it was returned to the scimitar vein (*arrows*) (**b**). Selective angiography of the anomalous artery (*arrowheads*) after the procedure. Cessation of the anomalous systemic arterial flow to the localized region of the right lower lobe was confirmed (*open arrows*) (**c**)

First, right pulmonary angiography was performed with a 7-Fr Berman angiographic balloon catheter (Arrow International, Inc., Reading, PA, USA) through the right femoral vein under local anesthesia. The right pulmonary artery was hypoplastic and was not distributed in the basal lung (Fig. 3a). It returned to the right pulmonary vein (scimitar vein) via the normal lung parenchyma (Fig. 3b). We then cannulated the anomalous artery that originated from the aorta near the right inferior phrenic artery using a 5-Fr catheter through the right femoral artery. Selective angiography revealed several thick and tortuous arteries localized in right basal lung, which was strongly enhanced (Fig. 4a), with flow returned to right pulmonary vein (scimitar vein) (Fig. 4b). No arteriovenous malformations such as an anomalous artery to the scimitar vein were observed. These anomalous arteries were suspected to be the most likely cause of the hemoptysis rather than occlusion or stenosis of the scimitar vein. Therefore, the proximal anomalous artery was embolized using 5 fibered interlocking detachable coils (Boston Scientific, Natick, MA); one 6 mm × 20 cm, one 10 mm × 30 cm, and three 14 mm × 30 cm, after the injection of a small amount of hand-cut gelatin sponge particles of approximately 2 mm in size.

**Fig. 5:**
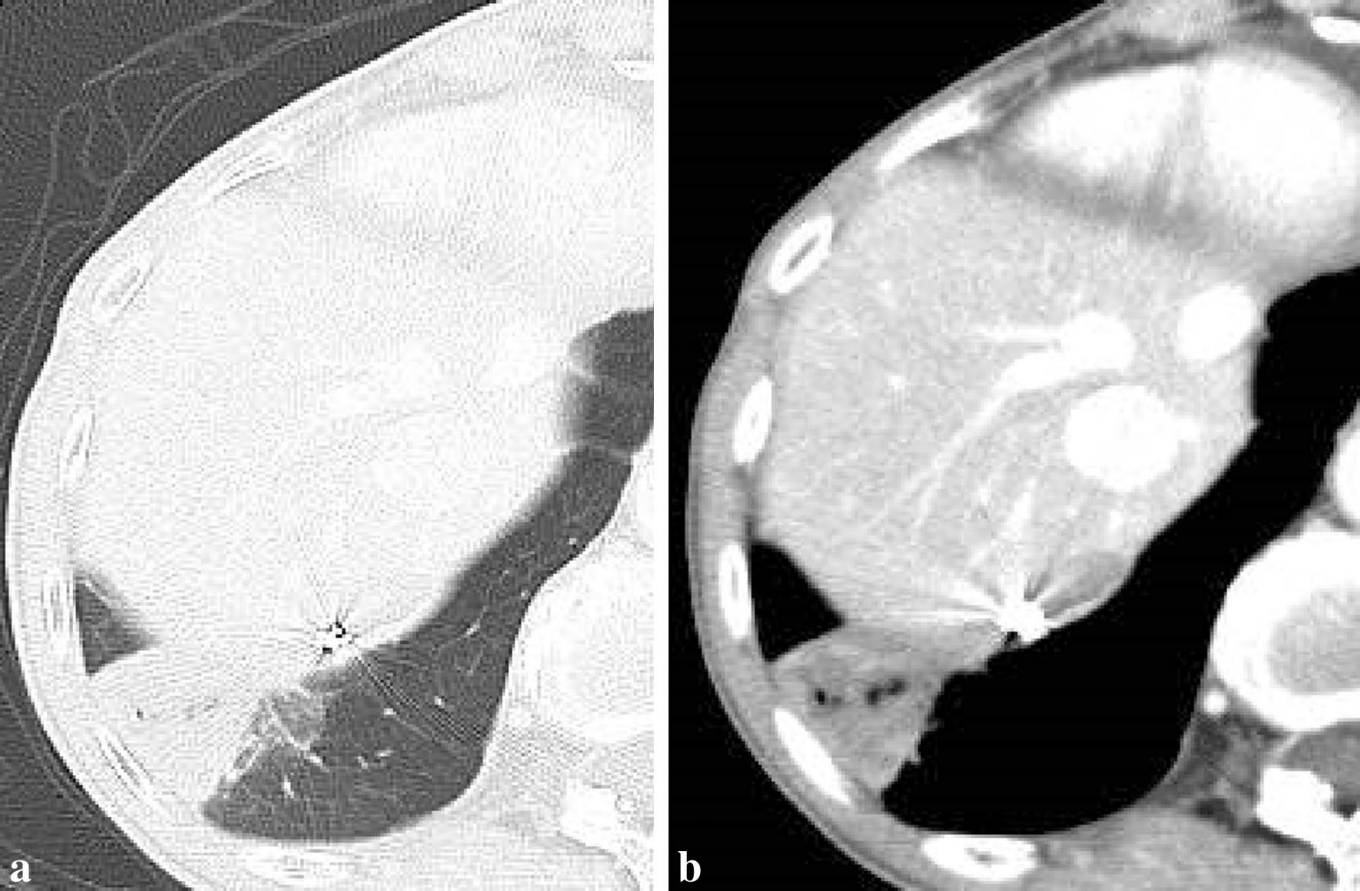
Contrast-enhanced computed tomography showed a localized wedge-shaped pulmonary opacity closely approximating the pleura, which indicated pulmonary infarction. **a** lung window image, **b** mediastinal window image

Afterwards, selective angiography showed occlusion of the anomalous systemic arterial flow to the localized region of the right lower lobe (Fig. 4c). After the procedure, the patient complained of a low-grade fever lasting for 10 days, and contrast-enhanced CT showed a localized wedge-shaped pulmonary opacity closely approximating the pleura, which was thought to indicate pulmonary infarction (Fig. 5a, b). During this period, the patient’s C-reactive protein level was slightly elevated at 2.5 mg/dL. Other laboratory findings (including leukocyte count, D-dimer, and LDH levels) were within normal limits. It resolved with conservative treatment, and no other complications were seen. She has not experienced any subsequent recurrence of the hemoptysis during a follow-up period of 6 months.

## Discussion and evaluation

We performed TAE for hemoptysis associated with anomalous systemic artery in a patient with scimitar syndrome. The distal portions of anomalous arteries were embolized using gelatin sponge particles and the proximal portion was embolized using fibered detachable coils. Although a small pulmonary infarction was observed as a complication, the patient has been well without other complications.

Scimitar syndrome can present with a wide clinical spectrum of symptoms either early in the neonatal period or later in life. Dupuis et al. (1992) divided scimitar syndrome into 3 main forms: an infantile form with symptoms and pulmonary hypertension, an “older” adult form distinguished by being asymptomatic in infancy, and a form with associated congenital cardiac anomalies. 3DCT-VR and multiplanar reformation offer an accurate means of noninvasive diagnosis that avoids cardiac catheterization and leads to a satisfactory classification of variant scimitar syndrome (Legras et al. 2012) as in our patient. Long-term follow-up of the adult form of scimitar syndrome without pulmonary hypertension shows that this condition is usually well tolerated, and close observation is recommended (Dupuis et al. 1992). However our patient’s symptom of progressive and recurrent hemoptysis was an indication for treatment. Cardiac catheterization and angiography were required for therapeutic decisions. Systemic circulation to the right basal lung, which had a higher pressure than the pulmonary circulation, was thought to be the most likely reason for the hemoptysis because pulmonary hypertension was excluded by hemodynamic measurements obtained by right heart catheterization. In describing the pathological findings of anomalous arteries, Huang et al. (2005) showed that there is a lack of muscular lamina in the vessel, which might explain unexpected fatal hemoptysis, and recommended treatment even for asymptomatic patients.

Surgery has been the standard therapy for symptomatic patients, and lobectomy was performed in most of the cases. Other operational procedures include segmentectomy, anastomosis between the anomalous artery and pulmonary artery, and ligation of the anomalous artery (Abe et al. 2011). Recently, a few case reports have discussed TAE of the anomalous systemic arterial supply as an alternative to surgical ligation in symptomatic patients with or without scimitar syndrome (Nedelcu et al. 2008; Jiang et al. 2011; Gümüştaş et al. 2013; Saida et al. 2006). Nedelcu et al. (2008) reported TAE of the anomalous systemic arterial supply as a suitable treatment for the patients with scimitar syndrome association that pulmonary and vascular surgeries are required for curative treatment.

Coils, Amplatzer vascular plugs, and N-butyl cyanocrylate were used in the most of previous reports. Gümüştaş et al. (2013) recommended proximal occlusion of the anomalous artery with coils or Amplatzer vascular plugs because embolization using particulate materials carries the risk of pulmonary ischemia. Abe et al. (2011) also recommended that use of particulate materials for embolization should be avoided to minimize the risk of pulmonary infarction. However, Izzillo et al. (2000) experienced tiny pulmonary infarctions even with coil embolization. Contrastingly, Anil et al. (2012) used N-butyl cyanocrylate without complication to solidify the coils for proximal embolization after creating a scaffold of metallic coils. We used a small amount of gelatin sponge particles of 2 mm in size, which resulted in a small pulmonary infarction.

Jiang et al. (2011) guessed that the low frequency of pulmonary infarction and the absence of ischemic complications following TAE are likely explained by the abundant available collateral circulation from the bronchial, intercostal, inferior phrenic, and other nearby arteries. However, Abe et al. (2011) noted that compensatory enlarged bronchial arteries may become collateral vessels to the affected lung and cause risk of recurrent hemoptysis. Moreover, Rubin et al. (1994) reported a case of fatal massive hemoptysis associated with anomalous systemic arterial supply to the basal lung by vulnerable anomalous artery breakdown. Delivery of a large amount of thin gelatin sponge particles may induce pulmonary infarction. The small amount of relatively large-sized (2 mm) gelatin sponge particles used in our case might not only reduce the risk of reperfusion to distal anomalous arteries from collateral vessels but also reduce the number of embolic coils needed. Therefore, we used gelatin sponge particles because we believe that their use to embolize distal arteries reduces the risk of recurrence. Although we experienced pulmonary infarction as a complication, the lesion was localized and conservative treatment was feasible, which caused no major adverse events in our patient. In fact, there are a few reports of complications after TAE of the anomalous systemic arterial supply, and these were treated with conservative management (Jiang et al. 2011). Additionally, in examining the possibility of such complications, it was certainly necessary that bronchial angiography be performed to evaluate the vascularity of the lung parenchyma. However, it might be better to avoid the use of gelatin sponge particles in patients with a similar anomaly without pulmonary artery distribution because of the possibility of causing severe pulmonary infarction.

## Conclusions

Hemoptysis in our patient with scimitar syndrome associated with anomalous systemic arterial supply to the basal lung was successfully treated with TAE. Although a small pulmonary infarction was observed as a complication, conservative management was possible. Because deformities of the blood vessels and the lungs are frequently complex in scimitar syndrome, patients treated with surgical repair of this disorder may be at higher risk than those treated less invasively. Therefore, we believe that TAE is safe and effective treatment for hemoptysis in patients with an anomalous systemic arterial supply to the basal lung, especially in patients with scimitar syndrome. However, it may be that the use of gelatin sponge particles should be avoid in patients with similar anomaly without pulmonary artery distribution, because there is a possibility of causing severe pulmonary infarction.

## Consent

Written informed consent was obtained from the patient for the publication of this report and the accompanying images.
